# High affinity binding of hydrophobic and autoantigenic regions of proinsulin to the 70 kDa chaperone DnaK

**DOI:** 10.1186/1471-2091-11-44

**Published:** 2010-11-08

**Authors:** Volker Burkart, Rahel K Siegenthaler, Elias Blasius, Koen Vandenbroeck, Iraide Alloza, Waltraud Fingberg, Nanette C Schloot, Philipp Christen, Hubert Kolb

**Affiliations:** 1 German Diabetes Centre, Leibniz Institute at Heinrich Heine University Düsseldorf, Institute of Clinical Diabetology, Auf'm Hennekamp 65, D-40225 Düsseldorf, Germany; 2 Department of Biochemistry, University of Zurich, CH-8057 Zurich, Switzerland; 3 Applied Genomics Research Group, McClay Research Center for Pharmaceutical Sciences, Queen's University, Belfast BT9 7BL, UK; 4 Current Address: Research Group Immunobiology, Heinrich-Heine-University Düsseldorf, D-40225 Düsseldorf, Germany

## Abstract

**Background:**

Chaperones facilitate proper folding of peptides and bind to misfolded proteins as occurring during periods of cell stress. Complexes of peptides with chaperones induce peptide-directed immunity. Here we analyzed the interaction of (pre)proinsulin with the best characterized chaperone of the hsp70 family, bacterial DnaK.

**Results:**

Of a set of overlapping 13-mer peptides of human preproinsulin high affinity binding to DnaK was found for the signal peptide and one further region in each proinsulin domain (A- and B-chain, C-peptide). Among the latter, peptides covering most of the B-chain region B11-23 exhibited strongest binding, which was in the range of known high-affinity DnaK ligands, dissociation equilibrium constant (K'd) of 2.2 ± 0.4 μM. The B-chain region B11-23 is located at the interface between two insulin molecules and not accessible in insulin oligomers. Indeed, native insulin oligomers showed very low DnaK affinity (K'd 67.8 ± 20.8 μM) whereas a proinsulin molecule modified to prevent oligomerization showed good binding affinity (K'd 11.3 ± 7.8 μM).

**Conclusions:**

Intact insulin only weakly interacts with the hsp70 chaperone DnaK whereas monomeric proinsulin and peptides from 3 distinct proinsulin regions show substantial chaperone binding. Strongest binding was seen for the B-chain peptide B 11-23. Interestingly, peptide B11-23 represents a dominant autoantigen in type 1 diabetes.

## Background

Prokaryotic and eukaryotic cells employ chaperones for guiding polypeptides during synthesis towards proper folding, for preventing misfolded proteins from aggregating, for re-establishing proper conformation or channeling misfolded polypeptides towards intracellular degradation. Under conditions of cell stress, such as heat stress or a high rate of protein synthesis, there is a higher amount of polypeptides misfolded, and in parallel there is a rapid increase of chaperone availability [1-4]. As one of the dominant members of the chaperone family, heat shock protein (hsp) 70 shows strong and preferential upregulation in various cell populations exposed to stress conditions [5]. Following the general principle of (poly)peptide chaperoning, hsp70 interacts with proteins by transiently binding to amino acid regions with distinct physicochemical properties. Detailed sequence analyses of hsp-chaperoned polypeptides identified stretches of at least seven amino acids with a core region of up to five hydrophobic amino acids as prominent binding motif for members of the hsp70 family [6,7]. Besides their function of (poly-)peptide guidance, chaperones induced by stress serve as danger antigens to the innate immune system [8-11], and those peptide regions of target proteins interacting with the peptide binding region of chaperones may be transferred onto MHC molecules. This mechanism has been termed re-presentation (of endogenous peptides to T helper cells) and may facilitate the induction of anti-tumor reactivity or the rise of autoimmunity [12-15].

Insulin is a primary product of protein synthesis of pancreatic β-islet cells. The peptide hormone is generated from the precursor forms preproinsulin and proinsulin. The mature, biologically active monomer of insulin is composed of an A- and B-chain; its structure is stabilized by intra- and inter-chain disulfide bonds.

Interestingly, insulin represents a dominant antigen during the development of the immunological processes leading to pancreatic β-cell destruction and (insulin-dependent) type 1 diabetes. Although the hormone is a primary target of autoantibodies that emerge early in the prediabetic phase [16] the stimulation of cell-mediated immune processes including the activation of insulin-reactive T-lymphocytes seems to be of major importance for the progression of β-cell-directed immune reactivity [17]. In fact, insulin-specific T-cells can be isolated from human subjects both in the prediabetic phase and the onset of type 1 diabetes and are present in the diabetes-prone non-obese diabetic (NOD) mouse, an animal model of the human disease [18,19].

In view of the chronic endoplasmatic reticulum stress conditions with enhanced chaperone activity observed for insulin producing pancreatic β-cells during islet inflammation in (pre) type 1 diabetes as well as during metabolic stress in states of insulin resistance and obesity [20,21], we hypothesized that (prepro-) insulin interacts with chaperones. In our experimental approach we therefore determined the ability of preproinsulin-derived 13-mer peptides, monomeric proinsulin or native insulin to bind to a chaperone. In the current study we used bacterial hsp70 as the best characterized member of the large and evolutionary well-conserved hsp70 chaperone family [22].

## Results

### Identification of DnaK binding peptide regions in preproinsulin

**Figure 1:**
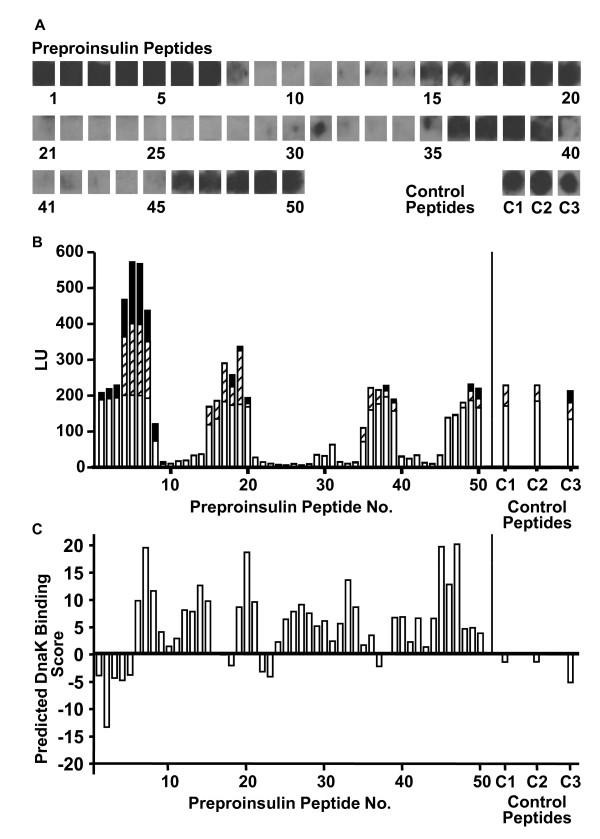
**Interaction between DnaK and immobilized preproinsulin peptides**. DnaK was allowed to bind to a cellulose membrane carrying 50 spots of overlapping 13-mer preproinsulin peptides and three spots of control peptides (C1, C2, C3) followed by electroelution and identification of eluted DnaK by specific antibody and chemiluminescence. **A**: The grey-scales of the spots correspond to the amounts of DnaK released from the peptide spots during the first elution cycle. **B**: Total DnaK released after the first (open bars), second (hatched bars) and third (solid bars) elution cycle, quantified by densitometry. The data are given in arbitrary luminescence units (LU). **C**: Probability scores for the binding of DnaK to preproinsulin peptides (and control peptides) as calculated by an algorithm designed to predict the affinity of 13-mer peptides to DnaK. Negative scores denote higher DnaK binding probabilities, positive scores denote low/no DnaK binding. A cut-off score of -5 would correctly predict 95% of the good binding sites. With scores above -5 the reliability of the prediction of binding decreases.

The interaction of the 70 kDa chaperone DnaK with proinsulin was investigated by analyzing the binding of the chaperone to immobilized 13-mer peptides covering the entire length of the unprocessed precursor of the hormone. Soluble DnaK showed differential affinities to the membrane-bound peptides. As indicated by the staining intensities of the spots in Figure 1A, four clusters of peptides covering four discrete regions of the preproinsulin molecule exhibited increased DnaK retention capacities in a range similar to that of the control peptides C1, C2 and C3 with well-documented high affinities to DnaK. One DnaK binding region was located in the signal peptide (peptides 1-8) and a second in the C-peptide (peptides 36-39), connecting the proinsulin A- and B-chain. Two further DnaK binding regions were located in the A-chain (peptides 46-50) and B-chain (peptides 15-20). Densitometric analysis of DnaK eluted from the immobilized peptides indicated peak binding to peptides 1-7, 17-19, 36-39 and 46-50 (Figure 1B). The application of an algorithm developed to predict the DnaK binding probability of peptides roughly confirmed the location of three of these binding regions in the preproinsulin molecule (signal peptide: peptides 1-5; B-chain: peptides 17 and 18; C-peptide: peptides 37 and 38) (Figure 1C). In contrast to the experimental findings, the algorithm did not predict DnaK binding for the A-chain region, but identified an additional DnaK binding region at the position of the peptides 22 and 23 which are located in a region connecting the B-chain and the C-peptide. As expected, the amino acid sequences of the control peptides yielded DnaK binding with scores in a range of -1.53 (C2) and -5.29 (C3).

**Figure 2:**
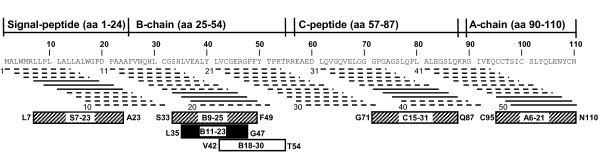
**Positioning of DnaK-binding peptides in preproinsulin**. Lines below the amino acid sequence represent the 50 immobilized 13-mer preproinsulin peptides used in the screening approach. Peptides with low or high DnaK-retention are shown as dashed or straight lines, respectively. Hatched boxes indicate the cores of the DnaK-binding regions as deduced from the 13-mer peptides showing peak values of bound DnaK. The solid black box indicates peptide B11-23 with high DnaK affinity and the open box indicates peptide B18-30 wit low DnaK affinity.

The overlapping peptides with peak binding of DnaK as determined in the solid phase assay were compared with the underlying peptide sequence of preproinsulin. The corresponding DnaK binding sequences in the preproinsulin molecule were amino acids S7-23 of the signal peptide, B9-25 of the B-chain, C15-31 of the C-peptide and A6-21 of the A-chain (Figure 2).

### Quantitative analysis of proinsulin peptide binding to DnaK

**Figure 3:**
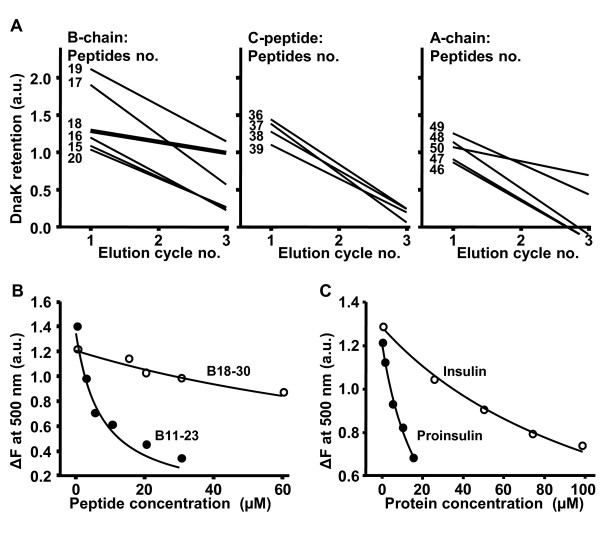
**Quantitative analysis of proinsulin peptide binding to DnaK**. **A**: For DnaK-binding peptides of the proinsulin A-, B-chain or C- peptide the amount of DnaK released during the three electroelution cycles was determined in order to assess the progress of DnaK dissociation from the peptides. For this analysis the strength of the signals obtained from the high affinity DnaK ligand C3 after each elution cycle (cycle 1: 134 LU; cycle 2: 46 LU; cycle 3: 33 LU) was set as "1" and used as reference for the signal strengths obtained from the investigated peptides after the corresponding cycles of elution. For each peptide, the resulting three data points (corresponding to the three elution cycles) were subjected to linear regression analysis and for better comparison of the progress of dissociation a line was plotted against the elution cycles. The bold line in the left panel indicates the release of the B-chain peptide 18. In a fluid phase competition assay a-NR (200 nM) and DnaK (1 μM) were incubated in the presence of increasing concentrations of peptide B11-23 (solid circles) or B18-30 (open circles) **(B) **or increasing concentrations of proinsulin (solid circles) or insulin (open circles) **(C)**. The difference in fluorescence emission intensity at 500 nm was plotted as a function of the concentrations of peptides or proteins.

Electro-elution of DnaK from immobilized peptides was performed in three consecutive cycles and the progress of peptide release was determined as an estimate of their DnaK affinity. The amounts of DnaK eluted in the first cycle (Figure 3A) correspond to the electroblots shown in Figure 1A. The amounts of DnaK released during elution cycles 2 and 3 were compared with the release of DnaK from the high affinity control ligand C3 (set as 1 in Figure 3A). Most peptides retained DnaK less than the control peptide during elution cycles 2 and 3 except for peptides 18 and 50. Of these two, peptide 18 exhibited better retention of DnaK indicating highest affinity in this assay (Figure 3A, left panel).

Interestingly, the peptide 18 (B11-23) corresponds to the insulin peptide B9-23, which had been identified as an immunodominant epitope of autoimmune T-cell reactivity in type 1 diabetes. Therefore, we analyzed the interaction of DnaK with peptide B11-23 also in a fluid phase assay. We determined the capacity of a peptide to compete with the fluorescent high-affinity DnaK ligand a-NR (dissociation equilibrium constant K'd = 0.41 ± 0.02 μM) for binding to DnaK [1]. Increasing concentrations of non-labeled peptide B11-23 resulted in a decrease of the fluorescence signal reflecting effective displacement of a-NR from DnaK by the peptide (Figure 3B), corresponding to an apparent dissociation equilibrium constant (K'd) of 2.2 ± 0.4 μM of the competing peptide B11-23. By contrast, peptide B18-30, which is an adjacent peptide from the proinsulin B-chain region but without significant DnaK-affinity, was much less effective in displacing a-NR (Figure 3B).

### Binding of DnaK to peptides versus intact insulin or monomeric proinsulin

Next, we used the fluid phase assay to analyze whether DnaK would interact with the intact insulin molecule. Native insulin, only poorly competed with a-NR (K'd 67.8 ± 20.8 μM) (Figure 3C, Table 1) indicating that the steric configuration of insulin oligomers prevents effective interaction with DnaK. Binding strength of insulin was in the range of the poorly binding peptide B18-30 (K'd 51.9 ± 12.8 μM). For comparison, the sigma32-derived control peptide, a high-affinity DnaK ligand, exhibited a dissociation constant of 0.5 ± 0.1 μM. The latter was in the same range as found for insulin peptide B11-23 (Table 1).

**Table 1:** Apparent dissociation equilibrium constants (K'_d_) of DnaK for insulin, proinsulin and peptides

Peptide/Protein (Sequence)	K'_d _(μM)
Human insulin (A- and B-chain)	67.8 ± 20.8
Peptide B18-30 (insulin B-chain aa18-30) VCGERGFFYTPKT	51.9 ± 12.8
Control peptide (sigma32 aa192-204) SHAMAPVLYLQDK	0.5 ± 0.1
Peptide B11-23 (insulin B-chain aa11-23) LVEALYLVCGERG	2.2 ± 0.4
Human proinsulin variant	11.3 ± 7.8

The DnaK affinity of insulin and insulin-derived peptides was determined in a competition assay as described in "Methods". Lines indicate the disulfide bonds in the insulin and proinsulin-molecules. The data show mean ± SD of three or four determinations.

Finally, we investigated the possible binding of DnaK to a proinsulin variant with decreased propensity to oligomer formation due to an inversion of the K-P sequence in the positions B28 and B29. This proinsulin variant interacted with DnaK at concentrations much lower than observed for insulin (K'd 11.3 ± 7.8 μM) (Figure 3C, Table 1).

## Discussion

Our experiments demonstrate that the 70 kDa heat shock protein DnaK is able to bind to 13-mer peptide segments of the preproinsulin molecule and that the binding is limited to four distinct preproinsulin regions, amino acids S7-23 of the signal peptide, B9-25 of the B-chain, C15-31 of the C-peptide and A6-21 of the A-chain. These peptide regions exhibit the leucine-rich motif found essential for binding of DnaK [23], usually a hydrophobic core of four to five residues enriched in L, but also in I, V, F and Y, and two flanking regions enriched in basic residues. Of the proinsulin molecule, strongest binding occurred to peptide 18 (B11-23), and this is probably mediated by a distinct hydrophobic motif formed by the three amino acid residues L-Y-L in the central positions B15, B16 and B17. This conclusion is supported by the finding that the B-chain-derived peptide B18-30, which comprises the six C-terminal residues of B11-23 but lacks the central L-Y-L sequence or a corresponding hydrophobic motif exhibited no significant DnaK affinity.

Native insulin exhibited only low affinity to DnaK. This observation indicates that steric hindrance prevents effective interaction with the chaperone, possibly due to the conformation of the polypeptide heterodimer or the aggregation of insulin molecules into dimers, trimers or hexamers, rendering the regions B11-23 and A6-21 less accessible. As deduced from the crystal structure of the insulin molecule http://swissmodel.expasy.org/repository, the B11-23 epitopes are buried at the interface between two mature insulin heterodimers, thus preventing access to the peptide binding site of DnaK. Similarly, the A-chain region A6-21 comprises a number of amino acid residues localized at the interfaces of the insulin oligomers. In particular, the surface-exposed E-residue in position A17 contributes to the stabilization of the dimer-dimer interfaces by its interaction with the F-residue in position 1 of the B-chain. Moreover, the C-residue in position A7 which is located at the surface of the insulin molecule, forms a stabilizing interchain disulfide bond with the C-residue in position 7 of the B-chain [24,25].

To further elucidate a potential impact of steric conditions on DnaK-insulin interactions we investigated the DnaK binding of a proinsulin variant with an inversion of the K-P sequence in positions B28 and B29 causing a decreased tendency to oligomerization. This variant indeed exhibited a much higher DnaK affinity than mature insulin. However, the Kd obtained for the mature insulin molecule may only represent an estimate for the (low) chaperone affinity of the hormone, as the fluid phase system applied in our approach is optimised for studies on the interaction of chaperones with peptides rather than larger proteins. Nevertheless, previous studies which applied experimental systems designed to investigate the binding of DnaK to polypeptides demonstrate weak, but significant interactions between DnaK and full-length, native proteins [26]. In those studies, DnaK binding was found to depend on the accessible hydrophobic area of a protein and was characterized by dissociation constants of 1 - 50 μM, a range comparable to the Kd of 67.8 ± 20.8 μM found for insulin in our current study. Taken together, these observation suggest that not only the (pro)insulin peptides but also mature proinsulin monomers can interact with DnaK and that there is little interaction between DnaK and native oligomeric forms of insulin.

In our current approach we selected insulin as the predominant β-cell specific (auto-) antigen. DnaK was selected as a representative member of the dominant and phylogenetically high conserved hsp70 family with the best characterized chaperone activity [27,28]. Although the members of the hsp70 family were found to exhibit some differences in peptide binding specificities [28] extensive comparative analyses revealed a common binding specificity for peptide stretches of at least seven amino acid residues with a hydrophobic core [7,28] thus largely resembling the hydrophobicity distribution pattern of the insulin-derived peptide B11-23. Therefore, our analyses on the interaction of potentially autoantigenic insulin peptides with the chaperone DnaK was performed in a suitable model system which only partially reflects the situation in vivo, in human patients. Further detailed studies of the interaction of insulin and proinsulin with human hsp70 isoforms will be needed to fully understand the rules of (pro-)insulin-chaperone interaction.

Besides the chaperoning function, one other property of hsp70 family chaperones is the ability to induce T-cell immune responses to complexed peptides [14,15,29-31], and a role of this pathway has been postulated for the induction of autoimmune disease [12,32,33]. It therefore is noteworthy that the three DnaK-binding regions of proinsulin, amino acids B9-25 of the B-chain, C15-31 of the C-peptide and A6-21 of the A-chain superimpose with the three major target regions for T-cell autoimmune reactivity in the NOD mouse model or in humans with β-cell autoimmunity [18,34-36]. In the mouse model, the B-chain region around B16 was identified as critical for disease development, i.e., mutating the native E in position B13 to Q abolished T-cell reactivity to the peptide B9-23 [34]. Moreover, replacement of Y to A in position B16 abrogated the response of B9-23 - reactive T-cell clones as well as the development of diabetes in vivo [37]. I.e., the peptide B11-23 is closely associated with diabetes pathogenesis, and its diabetogenic potential depends on an intact core sequence, comprising the region which is important for the binding of this peptide to DnaK.

As the insulin molecules with the mutated B-chains completely retain their metabolic activity [34,37] it has to be assumed that a single amino acid-exchange does not impair the formation of the correct tertiary structure, a complex process that most likely involves proper binding and assistance by chaperones. The findings from the mutated insulin molecules therefore further support the view, that the peptide binding motifs of chaperones are not determined by strictly defined amino acid sequences but by more roughly defined peptide properties, e.g. by the hydrophobicity distribution pattern within a stretch of amino acids [23]. In contrast, T-cell receptor-mediated peptide recognition is known to be a highly selective and largely amino acid sequence specific process. Based on these considerations it may be speculated that chaperones are able to bind defined sets of insulin peptides characterized by hydrophobic motifs. Chaperone-peptide complexes may be recognized by antigen presenting cells which perform the ultimate decision on the pathogenicity of a peptide by applying stringent, highly sequence-sensitive selection criteria during processing and re-presentation of the peptide in the context of MHC structures and co-stimulatory signals.

Further studies are warranted to analyze whether enhanced binding of proinsulin monomers or misfolded (pro)insulin to hsp70 family chaperones during periods of β-cell stress contributes to the pathogenesis of autoimmune diabetes.

## Conclusions

Our observations indicate that the hsp70 chaperone DnaK is able to bind to three discrete regions of the proinsulin molecule which are characterized by a central hydrophobic amino acid motif flanked by regions enriched for basic amino acids. Binding occurs to 13-mer peptides and to a proinsulin molecule modified to prevent oligomerization, but not to native insulin. The three DnaK-binding regions of proinsulin, aminoacids B9-25 of the B-chain, C15-31 of the C-peptide and A6-21 of the A-chain superimpose with the three major target regions for T-cell autoimmune reactivity in autoimmune diabetes.

## Methods

### Reagents

Purified recombinant DnaK was obtained from Stressgen, Victoria, BC, Canada. The 13-mer insulin peptides used to determine dissociation equilibrium constants were synthesized by the Peptide Synthesis Facility, Leiden University, Medical Center, Leiden, The Netherlands and the peptide NRLLLTG (NR, purity >95%) was synthesized by Chiron, Clayton, Victoria, Australia. Human insulin was provided by Novo Nordisk, Bagsvaerd, Denmark and proinsulin was provided by Lilly GmbH, Bad Homburg, Germany.

### Binding of DnaK to cellulose-bound preproinsulin peptides

A set of 13-mer peptides with an eleven amino acid overlap, covering the entire sequence of human preproinsulin (110 residues) and three control peptides with known high DnaK affinity (C1: WTYNAELLVLLEN (W436 - N448 from hemagglutinin); C2: GNTLVIVTADHAH (G382 - H394 from alkaline phosphatase); C3: SHAMAPVLYLQDK (S192 - K204 from sigma 32)) were synthesized in distinct spots on a cellulose membrane with an alanine-spacer at their COOH-terminus (Jerini Bio Tools GmbH, Berlin, Germany). For DnaK binding the peptide-carrying membrane was incubated for 60 min at 25°C with 100 nM recombinant DnaK in 31 mM Tris-HCl, pH 7.6, 170 mM NaCl, 6.4 mM KCl, 0.05% (v/v) Tween 20 and 5% (w/v) sucrose.

### Detection of DnaK retained by immobilized peptides

For the detection of peptide-bound DnaK the membrane was washed three times at 4°C with Tris-buffered saline (31 mM Tris-HCl, pH 7.6, 170 mM NaCl, 6.4 mM KCl) to remove unbound DnaK. Subsequently, peptide-bound DnaK was transferred onto polyvinylidene difluoride (PVDF) membranes by fractionated electroblotting in a semidry blotting unit at a constant power of 0.8 mA/cm^2^. The anode buffer contained Tris base and 20% (v/v) methanol and the cathode buffer (25 mM Tris base, 40 mM 6-aminohexanoic acid, 20% (v/v) methanol) was supplemented with 0.01 or 0.1% SDS. For the detection of transferred DnaK the PVDF membranes were incubated with a mouse monoclonal anti-DnaK antibody (Stressgen) for 90 min at room temperature followed by an incubation with a peroxidase-conjugated goat anti-mouse IgG antibody (Jackson Immuno Research Europe Ltd., Soham, UK) for 1 h at room temperature and the application of a chemiluminescence detection kit (ECL detection kit, Amersham Biosciences) [38]. The intensities of the resulting signals of the individual spots were quantified by densitometric evaluation using a Lumi-Imager workstation (Roche Applied Science, Mannheim, Germany) and expressed as Luminescence Units (LU) or used to determine the progress of DnaK release during sequential electroelution.

### Determination of dissociation equilibrium constants

DnaK was expressed and purified as described previously [39]. Peptide NR was labeled with the environmentally sensitive fluorophore acrylodan as described [2]. The dissociation equilibrium constants (K'd) of the complexes between DnaK and human insulin, proinsulin and selected 13-mer insulin peptides were determined by a competition binding assay with fluorescence-labeled NR peptide (a-NR). a-NR (200 nM) was incubated with 1 μM DnaK in the presence of increasing concentrations of competing substrate (human insulin, proinsulin or insulin peptide) at 25°C. After reaching equilibrium (~1.5 h), emission spectra were recorded from 400 nm to 600 nm (bandpass 4 nm) with a Perkin-Elmer spectrofluorimeter LS50B equipped with a stirrer and a thermostatically controlled cuvette holder. The excitation wavelength was set at 370 nm (bandpass 4 nm). Measurements were performed in a volume of 800 μl in 25 mM Hepes/KOH, 100 mM KCl, 10 mM MgCl_2_, pH 7.4 containing 1 mM DTT and 1 mM ADP-Pi at 25°C. K'd values were obtained from a least-squares fit of the difference in fluorescence emission intensity at 500 nm (ΔF) between a-NR in the presence and absence of DnaK as a function of the concentration of the competing substrate (human insulin, proinsulin or selected 13-mer insulin peptides) to the equation

(1) $$\Delta \text{F}=\Delta {\text{F}}_{\text{max}}\text{xP}/\left(\text{P}+\text{Kdx}\left(1+\text{L}/\text{K'd}\right)\right)$$

P denoting the concentration of DnaK, L the concentration of the competing substrate (human insulin, proinsulin or selected 13-mer insulin peptides), and Kd the dissociation equilibrium constant of the DnaK•a-NR complex in the presence of ADP and inorganic phosphate.

## Authors' contributions

HK and VB designed the experimental strategy for this study. VB, KV, IR and WF were involved in the experiments with immobilized peptides, RKS and PC conducted the DnaK-binding affinity studies. VB, EB, NCS and HK analyzed and interpreted the data, VB and HK drafted the manuscript and other authors made corrections to the manuscript. All authors read and approved the final manuscript.
